# Network Viscoelasticity from Brillouin Spectroscopy

**DOI:** 10.1021/acs.biomac.3c01073

**Published:** 2023-12-29

**Authors:** Raymundo Rodríguez-López, Zuyuan Wang, Haruka Oda, Metecan Erdi, Peter Kofinas, George Fytas, Giuliano Scarcelli

**Affiliations:** † Fischell Department of Bioengineering, University of Maryland, College Park, Maryland 20742, United States; ‡ School of Mechanical and Electrical Engineering, University of Electronic Science and Technology of China, Chengdu, Sichuan 611731, China; § School of Information Science and Technology, The University of Tokyo, Tokyo 113-8656,Japan; ∥ Department of Chemical and Biomolecular Engineering, University of Maryland, College Park, Maryland 20742, United States; ⊥ Max Planck Institute for Polymer Research, Ackermannweg 10, 55128 Mainz, Germany; # Institute of Electronic Structure and Laser, FO.R.T.H, N. Plastira 10, Heraklion, 70013, Greece

## Abstract

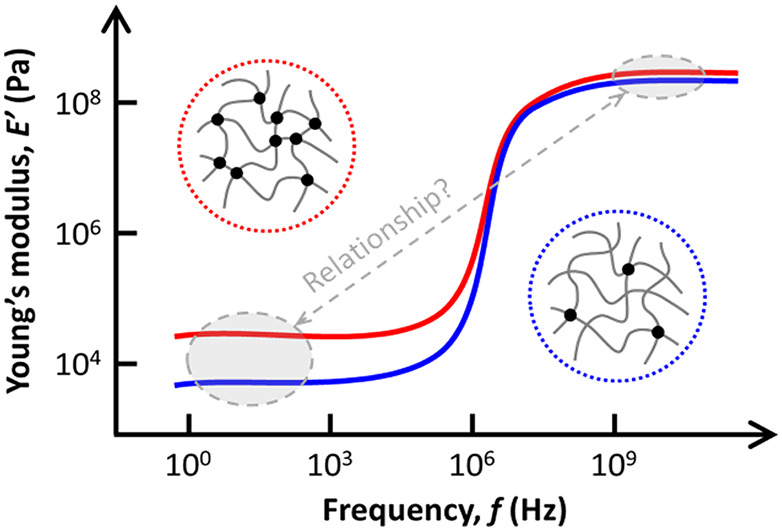

Even though the physical nature of shear and longitudinal moduli are different, empirical correlations between them have been reported in several biological systems. This correlation is of fundamental interest and immense practical value in biomedicine due to the importance of the shear modulus and the possibility to map the longitudinal modulus at high-resolution with all-optical spectroscopy. We investigate the origin of such a correlation in hydrogels. We hypothesize that both moduli are influenced in the same direction by underlying physicochemical properties, which leads to the observed material-dependent correlation. Matching theoretical models with experimental data, we quantify the scenarios in which the correlation holds. For polymerized hydrogels, a correlation was found across different hydrogels through a common dependence on the effective polymer volume fraction. For hydrogels swollen to equilibrium, the correlation is valid only within a given hydrogel system, as the moduli are found to have different scalings on the swelling ratio. The observed correlation allows one to extract one modulus from another in relevant scenarios.

## Introduction

**Figure 1:**
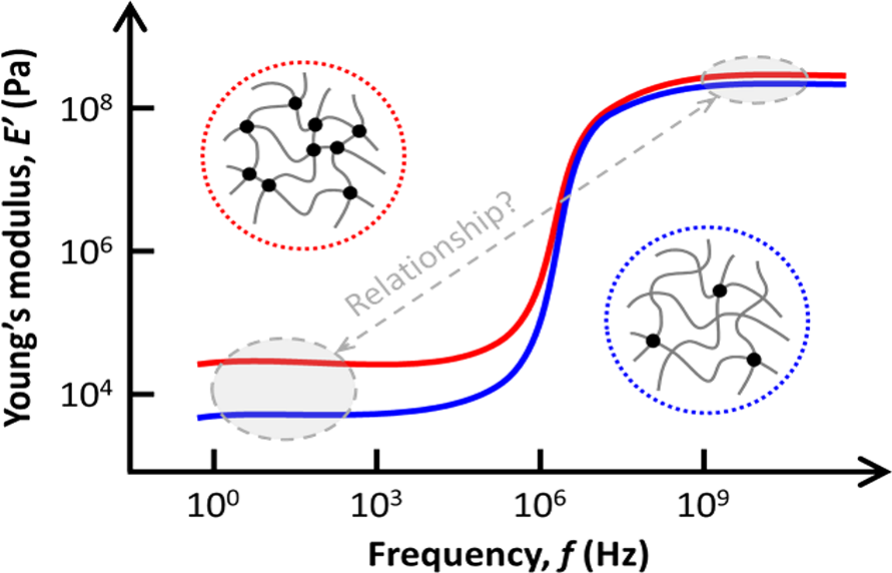
Schematic illustration of the frequency dependence of the elastic modulus in hydrogels. The low frequency plateau typically varies by about 2 orders of magnitude (from few kHz to 0.1 MHz), whereas at GHz frequencies, the modulus is about a few hundred MPa (for a Poisson’s ratio ∼ 0.48) with much smaller variations (∼30%) over the same composition range. The blue line denotes a hydrogel with a low cross-linked density, while the red line corresponds to a high cross-linked density hydrogel, with the latter displaying a higher elastic modulus than the former.

Biomechanical characterizations are traditionally performed by quantifying the Young’s (*E*) and shear (*G*) moduli in quasi static conditions (low frequencies < kHz), which are related through the Poisson’s ratio σ.^1^ However, the techniques to measure these moduli usually require contact forces (atomic force microscopy,^2^ micropipette aspiration,^3^ microrheology^4^) or lack resolution for a subcellular mapping in 3D (optical coherence elastography,^5^ ultrasound,^6^ magnetic resonance imaging^7^). Elastic moduli also have a strong frequency dependence, with values expected in the kPa range at low frequencies, but for sufficiently high frequency measurements, where the stress is applied fast enough to prevent the system from relaxing, the elastic response is found in the GPa range^8,9^ (Figure 1). In recent years, Brillouin light spectroscopy (BLS) has been demonstrated as a promising modality for biomechanical characterizations because it is all-optical and, thus, can provide noncontact, label-free, and high-resolution 3D mapping of the elastic properties of cells and tissues.^10−14^ However, BLS measures the longitudinal modulus (*M*) of materials at the hypersonic frequencies (GHz). Despite such fundamental differences, Young’s/shear and longitudinal moduli have been empirically correlated for several materials of biological interests, such as corneal^15^ and lens^11^ tissue, cells,^12^ or gelatin gels.^16^ The reason for this empirical correlation is a question that remains open in the field, and work is being done to understand if there is a fundamental reason behind the relationship between the elastic modulus measured at low frequencies and the elastic modulus measured at high frequencies in different systems. In this work, we use a polymer model system, polyacrylamide hydrogels, as a controlled system where the viscoelastic properties can be manipulated by changing the polymer volume fraction and cross-linking density of the hydrogel.

BLS has been previously used to study the viscoelastic characteristics of hydrogels and some of the variables that affect them, such as polymer volume fraction,^17,18^ temperature dependence,^19,20^ and cross-linking density.^21^ These studies showed that the longitudinal modulus can be affected by these intrinsic properties of the hydrogel, but without a comparison to any other viscoelastic characterization. On the other hand, different molecular weight PDMS hydrogels have been used to characterize the viscoelastic properties at low and high frequencies using rheometry and ultrasonic techniques to measure shear and longitudinal moduli, respectively.^22^ Shear and longitudinal moduli (measured by BLS) have been measured and correlated for hydrogels before, using gelatin^16^ and polyacrylamide^12^ or poly(ethylene oxide) gels.^23^ However, these studies only show an empiric correlation as a validation of the changes of viscoelastic characteristics of the hydrogel^12,16^ or use highly hydrated hydrogels as the object of study, where water content (>90%) strongly affects BLS measurements.^23^ The aim of this study is to investigate the underlying reasons behind such correlations with theoretical and experimental evidence on polymer model systems; in the process, we establish, quantitatively, the material and experimental conditions under which the correlation between the two moduli is valid or needs additional information. Furthermore, we cover a wider percentage of water, ranging from highly hydrated hydrogels with more than 95% water content to samples with ∼75% water content, a value closer to physiological conditions of tissue^24^ and cells.^25^

We hypothesize that the correlation originates from a common dependence of the two moduli on the same underlying local physicochemical properties.^12^ To elucidate the physical origin of the empirical correlation between shear and longitudinal moduli, we use polyacrylamide hydrogels (AA) with polymer volume fractions in the range of 5–20% as model systems. We show that both moduli depend on the local segmental packing of these hydrogels, expressed in an effective gel composition as the volume fraction that the polymer occupies in the hydrogel. Experimentally, this parameter depends on different characteristics of the hydrogel, such as the polymer–solvent interaction, water content, and cross-link density. In our AA samples, we analyze the dependence of the experimental shear and longitudinal moduli on the polymer volume fraction (ϕ), estimated experimentally. For shear modulus *G*, the expected strong ϕ-dependence is rationalized by an effective volume fraction (ϕ_eff_) based on the scaling theory of polymer networks that accounts for monomer–solvent interactions. In the case of the longitudinal modulus *M*, there is a much weaker ϕ-dependence due to the absence of polymer chain effects, and it is expected that the material obeys a linear or the inverse rule of mixtures.^26^ The latter was found to represent the experimental *M*(ϕ) of the AA, in agreement with the *M*(ϕ) of physical AA networks. Based on these considerations, we established the expected correlation between shear and longitudinal moduli and experimentally verified it with hydrogels prepared at three different conditions (neutral, positively charged, and an alternative initiator hydrogel) with tunable mechanical properties (by varying the cross-linker and polymer concentrations). We applied a similar workflow to the same AA hydrogels but swollen-to-equilibrium, where the equalization of the osmotic and elastic forces leads to different gel strand conformation.^27^ In this scenario we find that shear and longitudinal moduli are affected differently by the swelling ratio, which represents the amount of water that entered the hydrogel. As a result, important corrections to the empirical correlations must be applied.

## Materials and Methods

### Hydrogels: Preparation of the Relaxed and Swollen Hydrogels and Measurements of Their Polymer Volume Fractions

All chemicals used for the hydrogel synthesis were purchased from Sigma-Aldrich. Stock solutions w/v of 40% acrylamide as monomer and 2% *N*′,*N*′-methylene bis-acrylamide as cross-linker were prepared with DI water at room temperature and used with the desired amount of initial polymer volume fraction of monomer and cross-linker for different stiffness values.^28^ Tetramethylethylenediamine (TEMED) and a 10% ammonium persulfate (APS) solution prepared fresh were used as initiators for the neutral and charged gels. Hydrogen peroxide and temperature were used as initiators in the alternative initiator hydrogel option,^29,30^ following a protocol to form low molecular weight polyacrylamide chains. 3-Methacrylamidopropyltrimethylammonium chloride (MAPTAC) was used as the cationic monomer in the charged gels.^31^

For our experiments, a mixture of the desired amount of monomer and cross-linker were mixed with DI water. Afterwards, TEMED was added carefully into the mix (4 μL per mL), and last, APS was added into the mix (6 μL per mL).^28^ For the charged hydrogels, 1% of the MAPTAC was added to the mix and considered for the desired amount of monomer calculation. For these hydrogels, the pH level was adjusted with 0.1 M NAOH or 0.1 M HCl to pH 7 to allow polymerization.^31^ In the alternative initiator hydrogel, the mix was prepared in the same way as the neutral hydrogels, but the polymerization was carried out by adding 10 μL per mL of hydrogen peroxide and placing the mold in a temperature bath at 70 °C until polymerization was completed (adapted from refs (29 and 30)). For all hydrogels, the mix was poured into a mold to obtain a flat layer of hydrogel (<2 cm), and after letting the mix rest for 30 min to ensure full polymerization, a 5 mm diameter disk was cut and immediately used for measurements. If hydrogels were not used at the moment, they were maintained in a sealed mold when they were not being used to avoid gain or loss of water. Hydrogels were used in three different states: just after a polymerization, “relaxed state”; after swelling to equilibrium, “swollen state”; and after leaving the hydrogels to completely dry, “dry state”. The mass of the hydrogel for the “relaxed state” was measured just before the mechanical measurements. The “swollen state” hydrogels were obtained by immersing the hydrogel in DI water for 48 h, until the maximum amount of water was absorbed, and the mass of the hydrogel did not change anymore. The mass of the hydrogel in the “swollen state”, *W*_s_, was weighted. Afterward, the samples were left to dehydrate for >48 h, until only the polymer fraction was left (dry state) and weighted again to obtain *W*_d_. The volume fraction of polymer of the swollen hydrogels was calculated , is obtained from the volume of the swollen gels, *V*_s_. In view of the tiny amount of cross-linkers, they were neglected in the volume fraction calculations.

### Shear Rheology

The shear modulus *G* was measured by rheometry (TA Ares-G2 rheometer) with an 8 mm diameter parallel plate, with sandpaper added to the parallel plate to prevent the sample from slipping. A frequency sweep in the range of 0.1–10 Hz was performed in the linear viscoelastic region, taking the value of the storage shear modulus at 1 Hz and at 1% strain at 25 °C.^12^ All the values reported for shear modulus are the elastic or storage modulus *G*′, since this value is significantly greater than the loss modulus in this elastic region. The subscripts refer to the state of the hydrogel: *G*_r_ for relaxed hydrogels and *G*_s_ for swollen hydrogels.

### Brillouin Light Spectroscopy (BLS)

BLS is based on the interaction between light and acoustic waves or phonons within matter, leading to a frequency shift (*v*_B_) between the incident and scattered light. This frequency shift is related to the longitudinal modulus of the material:
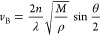
where *n* and ρ are the refractive index [*n* = 1.45 for monomer acrylamide^32^] and the density of the material [ρ_*p*_ = 1.13 g/cm^3^ for monomer acrylamide^33^]; λ is the wavelength of the incident light; *M* is the longitudinal modulus of the sample; and θ is the angle of the incident light. In this case, using a backscattering configuration, where θ = 180°, , and the formula becomes
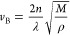
This means that the longitudinal modulus *M* can be calculated from the measured frequency shift . The frequency shift was measured using a Brillouin microscope set up similar to the one used in previous studies.^10,14,15,34^ An inverted confocal microscope, with a 660 nm continuous wave laser (Torus, Laser Quantum) with a power ∼30 mW was focused onto the sample by an objective lens (40×/0.6NA, Olympus), with a transverse resolution of ∼0.4 μm and an axial resolution of ∼2.6 μm. The backscattered light was collected by the same objective and coupled into the Brillouin spectrometer, composed of two-stage virtually imaged phase array (VIPA) etalons. A built-in house program (LabVIEW) was used to control the translational stage where the sample was placed, and a cross-sectional image of the center of the hydrogel was imaged in an *XZ* plane, obtaining an image with an X displacement of 500 μm and the entire hydrogel in Z representing the Brillouin shift of the hydrogels. An average of the whole scan was chosen as the representative value of the Brillouin shift for the measured hydrogel. The resolution of the image was chosen to be 20 × 50 μm per pixel; the image acquisition (EMCCD Andor camera) was performed with an exposure time of 50 ms. The subscripts refer to the state of the hydrogel: *M*_r_ for relaxed hydrogels and *M*_s_ for swollen hydrogels.

## Results and Discussion

### Relaxed Hydrogels

Longitudinal and shear moduli were measured for the different AA hydrogels in a “relaxed state”, which means just after polymerization and without any drying or swelling. The different hydrogels are labeled neutral charged gels (●), 1% cationic charged gels (▲), and low molecular weight hydrogels (★). They are prepared with initial monomer concentrations of 5% (black), 7.5% (green), 10% (blue), 15% (light blue) and 20% (red) monomer, each at four different cross-linking agent concentrations (ratio cross-linker 1:polymer = 1/*x*, with *x* = [30, 25, 20, 10]), as represented by the distinct data points. For the 15% and 20% hydrogels, only the 3 lower cross-linker ratios were used, since the higher cross-link ratios led to opaque hydrogels. All investigated hydrogels were clear and completely polymerized for measurements. The aforementioned color and figure code is maintained throughout this manuscript.

**Figure 2:**
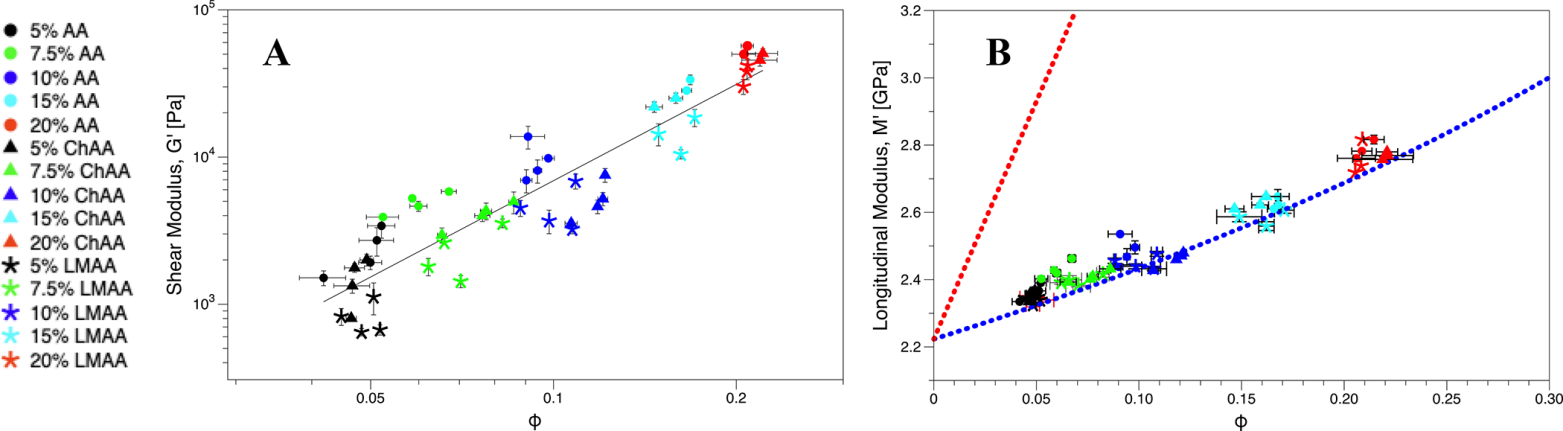
Shear and longitudinal moduli vs polymer volume fraction ϕ. The monomer concentration and hydrogel type are represented by color and shape, respectively. Different points with the same color and shape represent different percentages of cross-linker. (A) Log–log plot of shear modulus *G*_r_ vs experimental polymer volume fraction ϕ of AA hydrogels in relaxed state. The solid line represents *G*_r_ ∼ ϕ^2.2±0.1^, *R*^2^ = 0.85. (B) Longitudinal modulus *M*_r_ vs experimental polymer volume fraction ϕ of the same hydrogels. The red and blue dotted lines respectively denote the predictions by the linear and inverse laws of mixtures, using *M*_p_ = 16.35 GPa and *M*_w_ = 2.22 GPa. In both A and B, the data points are scattered for the different cross-linking agent contents at a constant AA concentration.

The nominal polymer volume fraction of the hydrogels ϕ (i.e., water content) was calculated as
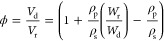
1where *V*_d_ and *V*_r_ are the volume of the dry and relaxed gels; ρ_p_ and ρ_s_ are the density of the polymer and the solvent (water in this case); and *W*_r_ and *W*_d_ are the mass of the gels in the relaxed and dry state (, where ρ_r_ = ρ_s_(1 – ϕ) + ρ_p_ϕ). All of the samples were weighted in the relaxed state (*W*_r_), and their longitudinal and shear moduli were measured right after. From the log–log plot of the shear modulus of the relaxed hydrogels *G*_r_ vs the nominal polymer fraction ϕ (eq 1) in Figure 2A, an overall scaling behavior (i.e., *G*_r_ ∼ ϕ^α^) with a slope α ∼ 2.2 is observed; separating the samples in labeled neutral charged gels (●), 1% cationic charged gels (▲), and low molecular weight hydrogels (★), the slope α increases from 2 to 2.5. The longitudinal modulus of relaxed hydrogels *M*_r_ is also plotted against ϕ (Figure 2B), and the inverse rule of mixtures (, where solid and liquid parts are weighted by their respective volume fractions; *M*_w_ is the longitudinal modulus of water and *M*_p_ is the longitudinal modulus of the pure polymer), represented by the blue dashed line, is found to represent the data in contrast to the linear rule of mixtures (*M*_r_ = ϕ*M*_p_ + (1 – ϕ)*M*_w_) denoted by the red dotted line. However, significant scattering around the fitting lines is observed in both *G*_r_(ϕ) and *M*_r_(ϕ). Although care was taken to ensure that measurements were done immediately after polymerization, there might be variability in water content: in a higher humidity day water enters through the atmosphere into the gel, whereas on a drier day the water in the gel may evaporate. Therefore, the water content in any state, particularly the “relaxed” state which is not an equilibrium state, may differ from the expected value. Since the experimental uncertainties of both moduli cannot account for the large deviations, the water content of the samples probably does not represent the driver of either hydrogel moduli. Indeed, when measuring ϕ, only the density and weight of the components of the mix are considered. To correctly evaluate the material response, other parameters such as the polymer–solvent interaction or cross-link density should also be taken into account.^35^ Among all studied samples, *M*_r_ in GPa changes by ∼21%, whereas the corresponding change in *G*_r_, falling in the kPa range amounts to ∼88 times. This indicates a much weaker variation in *M*_r_ than in *G*_r_, due to the much stronger volume fraction dependence of the latter.

Therefore, it is probably useful to correctly estimate an effective volume fraction (ϕ_eff_), which includes the polymer/solvent interaction and cross-link density, as defined by Rubber Elasticity Theory (RET) and the scaling concepts of semidilute polymer solutions. According to RET the shear modulus *G* is defined as^36,37^
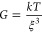
2where the mesh size, ξ = *bN*^*v*^, is the average linear distance between two adjacent cross-links, with *b* and *N* being, respectively, the Kuhn segment length and the number of Kuhn segments between two neighboring cross-links, and *v* is the scaling exponent defined by the polymer solvent interaction.^37^

Since the effective volume fraction is defined as^38^, the shear modulus of these hydrogels in a relaxed state depends on ϕ_eff_ as,
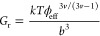
3

In Figure 2A, the observed scaling *G*_r_ ∼ ϕ^∼2.2^ indicates a good polymer–solvent interaction with an exponent ∼2.25 .^38^ Thus, we assumed good polymer–solvent interactions ()^39^ in eq 3 and obtained .

**Figure 3:**
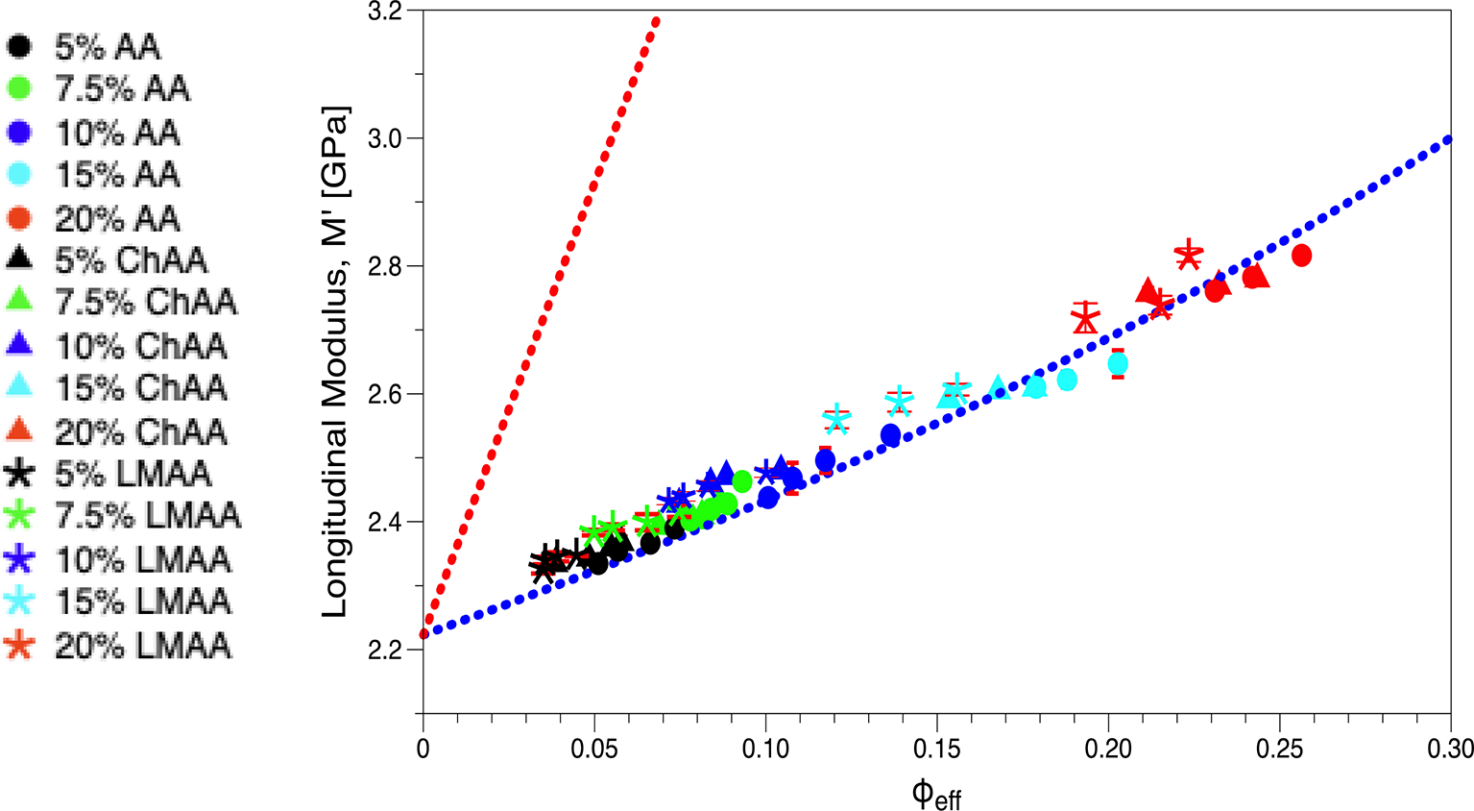
Longitudinal modulus *M* vs effective polymer volume fraction ϕ_eff_ of AA hydrogels in relaxed state. *M*_p_ = 16.35 GPa and *M*_w_ = 2.22 GPa are fixed values in the representation of *M*(ϕ_eff_) by eq 4 (blue dotted line). The red dotted line denotes the linear dependence of *M*(ϕ_eff_).

The longitudinal modulus of the physical networks of polyacrylamide semidilute solutions in Figure S1, is found to follow the inverse law of mixtures,^23,26^ meaning that the gel is a “biphasic” medium where the longitudinal modulus of the solid and liquid parts are inversely weighted by their volume fractions.
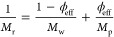
4where *M*_p_ = 16.35 GPa for the bulk AA polymer^23^ and *M*_w_ = 2.22 GPa for water following a previously reported behavior of the longitudinal modulus of the AA hydrogels.^23^ For the polyacrylamide physical network, see Figure S1, ϕ_eff_ = ϕ is the unique AA volume fraction, where it is observed that it follows the inverse law of mixtures, eq 4. The line width of the Brillouin spectrum also presents a slight increase with the polymer volume fraction as expected, but far from the transition from the liquid to solid phase^40,41^ (Figure S2). For the AA hydrogels in Figure 2, the concurrent representation of shear and longitudinal moduli by eqs 3 and 4, respectively, involves the Kuhn segment length *b* as an adjustable parameter. Using *b* = 1.5 nm for good solvency (eq 3) significantly improves the fitting quality of *M*_r_(ϕ_eff_; Figure 3). Note that this value of *b* is typical for flexible polymers like poly(methyl methacrylate).^42^ Thus, by assuming good polymer–solvent interactions for the hydrogels and the inverse law of mixtures, the scaling of the system and the Kuhn segment length are justified in a consistent way. Both *G*_r_ and *M*_r_ can be related to an effective polymer volume fraction, ϕ_eff_, which is an intrinsic property of the gel that dictates the trends of both moduli.

**Figure 4:**
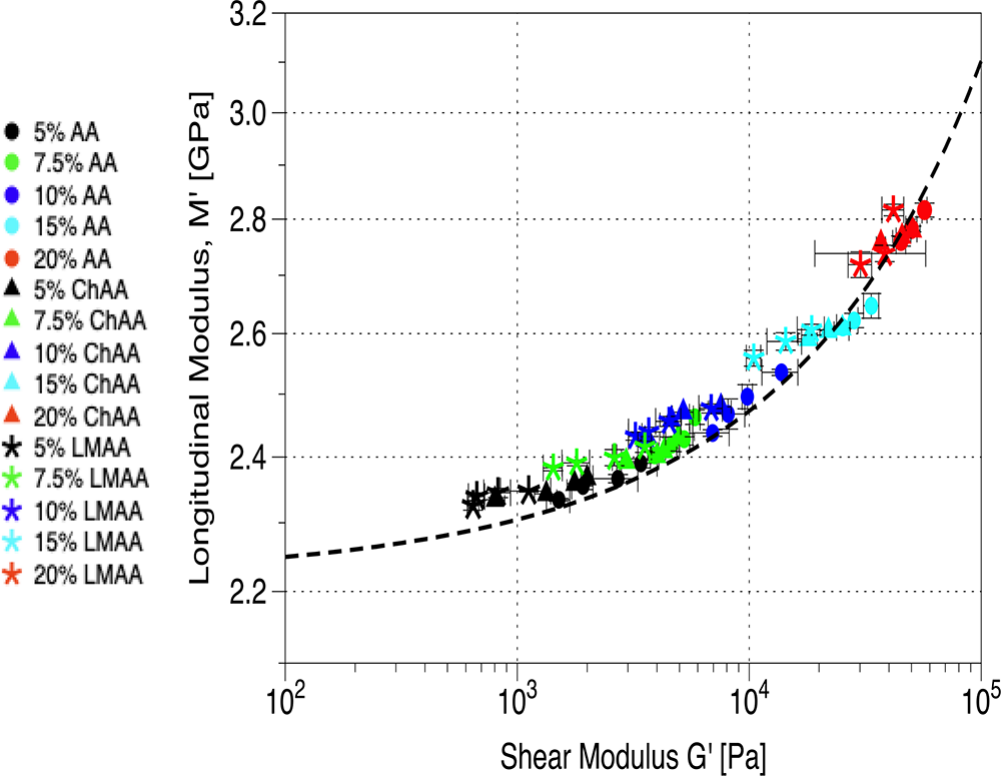
Longitudinal modulus vs shear modulus. Log–log plot of *M*_r_(*G*_r_) of the experimental moduli (symbols) and computed correlation (dashed line, eq 5) between the two moduli.


Hence, the correlation between the longitudinal *M*_r_ and shear *G*_r_ moduli shown in Figure 4 is represented (dotted line) by eq 5, derived by combining eqs 3 and 4.

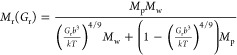
5This demonstrates that despite their different physical origins, both shear *G*_r_ and longitudinal *M*_r_ moduli depend on the effective polymer volume fraction, an underlying intrinsic property of the considered hydrogels. They can be correlated and predicted from each other with the necessary assumptions, namely, solvency and Kuhn segment length. The inverse law of mixtures (eq 4) adequately represents the longitudinal modulus of both physical (entangled) and chemical (cross-linked) AA hydrogels.

### Swollen Hydrogels

To obtain swollen-to-equilibrium hydrogels, the hydrogels in the relaxed state were left immersed in DI water for >48 h, until the weight did not change over time. Afterward, the *M* and *G* moduli were measured in the same way as the relaxed hydrogels condition. The swelling ratio,^38^*Q*, was also calculated for each condition of the hydrogels using the weight of the swollen and the relaxed hydrogels, Weight_S_ and Weight_R_, respectively: . Some changes in the system were expected, since the hydrogel structure adopts a different force-balance, explained by a different osmotic pressure in the system,^23,38,39^ and the swollen polymer network hypothesis links different theories to understand their behavior.^43^ Therefore, we should anticipate an impact on the relation *M*(*G*) employing the same three different groups of hydrogels.

**Figure 5:**
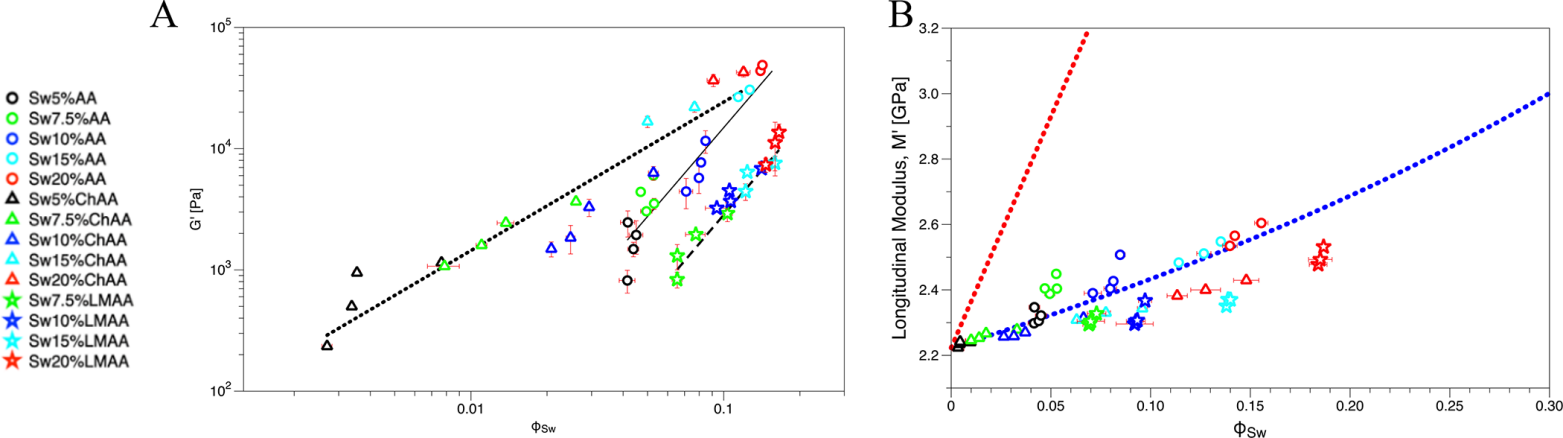
Shear and longitudinal moduli vs polymer volume fraction ϕ_Sw_. (A) Log–log plot of the shear modulus *G*_s_ vs ϕ_Sw_ of the AA hydrogels that are swollen to equilibrium. Each condition of the hydrogels shows distinct behavior. The slopes obtained from the power law representation for the swollen neutral, swollen cationic charged condition, and swollen low molecular weight conditions are 2.43 (solid line), 1.22 (dotted line), and 2.44 (dashed line), respectively. (B) Longitudinal modulus *M*_s_ vs ϕ_Sw_ of hydrogels swollen to equilibrium exhibiting large scattering of the data points. No trend following the inverse rule of mixtures can be assumed.

Following the same workflow as for the relaxed state hydrogels, both longitudinal and shear moduli are plotted as a function of a nominal volume fraction  in Figure 5, where *V*_d_ and *V*_s_ are the volume of the dry and swollen hydrogels; ρ_p_ and ρ_s_ are the density of the polymer and the solvent (water in this case); and *W*_d_ and *W*_s_ are the mass of the hydrogels in the dry and swollen state. The same color and figure codes are kept, except for using hollow symbols to distinguish the swollen state from the relaxed state. The lack of superposition of the measured shear *G* vs ϕ_Sw_ can be observed in Figure 5A, including the clear separation of the fits for the different groups suggests that the gels exhibit dramatically different behaviors already in traditional measurements. Due to the different behaviors, it is not possible to assume the same polymer interaction between groups as it was done before with relaxed gels. Also, a poor inverse rule of mixture law representation of the longitudinal modulus *M* is observed in Figure 5B, contrary to the behavior of the relaxed hydrogels (Figure 2B). The values of the longitudinal modulus and the polymer volume fraction are lower, and the apparent dependence with each other is lost. Thus, to understand the distinct behavior of the moduli in the swollen state (Figure 5), where the hydrogels reach equilibrium, we proceeded with their analysis separately.

For a polymer network swollen without any constraint, the network undergoes uniform swelling by the same amount in all directions (i.e., an affine deformation), and the swelling ratio at the macroscopic level of the gel is equivalent to the deformation  at the scale of each chain.^44^ The Young’s modulus (*E*)
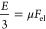
6is proportional to the density of the network strands μ ∼ *Q*^–1^ and the elastic free energy of a network strand ,^38,44^ where *R*_0_ and *R*_s_ are the mean-squared end-to-end distance of each chain in the unperturbed state and its fluctuation, respectively; in the low-swelling regime *R*_0_ ∼ *R*_s_.^38,44^ Hence,  and since the Young’s and shear moduli are proportional to each other for a given Poisson’s ratio of the material,^44^
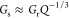
7where *G*_r_ denotes the shear modulus of the gel in the relaxed state.

**Figure 6:**
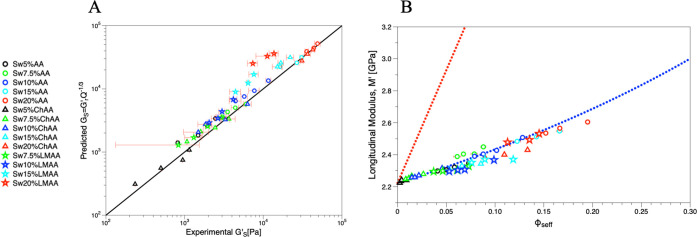
Shear and longitudinal moduli for swollen hydrogels. (A) Comparison of the shear modulus measured in relaxed gels *G*_r_ affected by the swelling ratio, vs the actual shear modulus measured on these gels *G*_s_. The black line represents *G*_s_ = *G*_r_*Q*^–1/3^. (B) The behavior of *M*_s_ seems a better fit following the inverse law of mixtures when considering ϕ_Seff_ = (ϕ_eff_ × *Q*^–1^).

Figure 6A shows the relationship of eq 7 (solid line) in a log–log plot of the experimental quantities, where it is observed that the scaled shear moduli of the hydrogels in the relaxed state affected by the swelling ratio () correlate well with the shear moduli *G*_s_ in the swollen condition for these hydrogels. Apart from some deviations for the 20%AA samples, the scaling is considered satisfactory within experimental errors and in view of the lack of scalability in Figure 5A; a higher *Q* would be needed to improve the scalability of hydrogels at higher compositions.

Turning to the longitudinal modulus, since it is affected only as a combination of the mixture, meaning the amount of polymer and solvent, the modulus should be represented as the volume-fraction-weighted average of both compositions (i.e., the Wood’s law). Here, the amount of water in the system affects the ϕ_eff_ and allows the introduction of an effective polymer volume fraction for the swollen gels, ; this ϕ_Seff_ compares well with the experimental polymer volume fraction ϕ_Sw_ (Figure S3). With Figure 6B it can be observed that the system still follows the inverse law of mixtures relation (eq 4), and ϕ_Seff_ is found to represent the experimental modulus *M*_s_ of the swollen hydrogels. Note that the fit without any adjustable parameter is less satisfactory than for the relaxed gels (Figure 2A), but much better than that using ϕ_Sw_ (Figure 5B).

To check the sensitivity of the representation in terms of the inverse rule of mixtures law for the experimental longitudinal modulus *M* to the material system, we compared the measured ϕ_Sw_ with ϕ_Seff_ that provides a good description as shown in Figure S4. It is partially the statistical deviation around the diagonal in Figure S4B that accounts for the quality of the *M*(ϕ_Seff_) fit in Figure 6B. This notion is also corroborated by the comparison of the experimental *M*_s_ with the predicted *M*(ϕ_Seff_) from the inverse law of mixture using ϕ_Seff_, as shown in Figure S5. The attempted representation of *M*_s_(ϕ_Seff_) by inverse law of mixtures is based on the successful description of *M*_r_(ϕ_eff_) in the relaxed state by eq 4. However, in contrast to the latter, *M*_s_(ϕ_Seff_) depends not only on ϕ_eff_, but also on *Q* (Figure S6A). In addition, the expected decrease of ϕ_Seff_ with *Q* is system dependent (Figure S6B). Using eq 4 the dependence of *M*_s_ in the swollen state can be written as a function of *Q* and *M*_r_(ϕ_eff_):
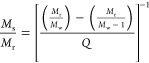
8The representation of the experimental ratio, (*Q*), does not lead to a superimposition for the three hydrogels (Figure S7) due the different swelling ratio of the relaxed hydrogels at similar ϕ_eff_ (Figure S6). But by However, by adjusting the ratio *M*_r_/*M*_w_(ϕ_eff_), a prediction for the *M* can be made (Figure S7). Thus, both *M*_s_ and *G*_s_moduli are affected by ϕ_eff_ of the hydrogels prior to swelling, but the longitudinal modulus *M*_s_(ϕ_Seff_) depends on the swelling ratio *Q*, while the shear modulus *G* depends on the volumetric deformation () of the network. Being able to predict these moduli separately, their mutual relationship for the swollen hydrogels can be similar to eq 5 for the relaxed hydrogels, by replacing  with
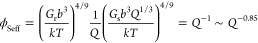
9Unlike eq 5, *M*_s_ is not only a function of *G*_s_, but also depends on *Q* according to eq 9, due to the hydrogel specific swelling, i.e., system-dependent ϕ_Seff_(*Q*).
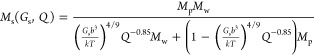
10

**Figure 7:**
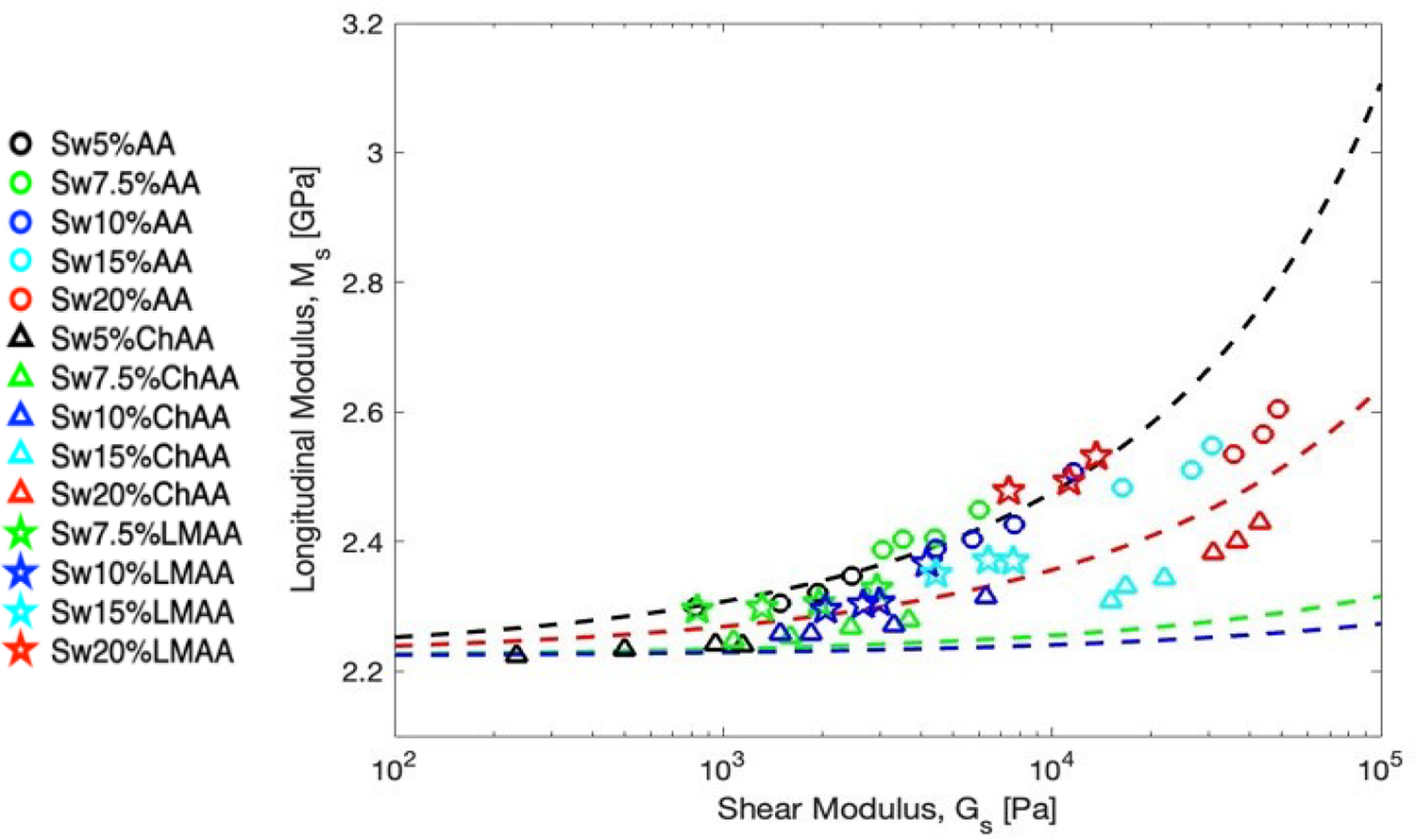
Prediction of the relationship for swollen hydrogels. Log–log plot of experimental *M*_s_ vs experimental *G*_s_. Symbols represent the experimental data, while the theoretical prediction of eq 9 assuming different swelling ratios for all the samples is represented with dashed lines [*Q* = 1 (black, for the relaxed state); *Q* = 2 (red); *Q* = 10 (green); *Q* = 20 (blue)]. The experimental data do not follow the same trend because the samples have different *Q* values.

Based on eq 10, the relationship between *M*_s_ and *G*_s_ for hydrogels in the swollen state requires additional information on the preceding relaxed state, expressed in their swelling ratio *Q*. Figure 7 displays the experimental *M*_s_(*G*_s_), as well as different lines that the systems would follow if all the samples had the same *Q* values (*Q* = 1, 2, 10, 20; represented by different dashed line colors). At relaxed state all samples follow the behavior of *Q* = 1, but since *Q* changes for the different hydrogels, there is not a single behavior that can be followed for the swollen hydrogels. It is easy to see that the successful superposition obtained in Figure 4 for the relaxed gels is not assured for the corresponding swollen state, and as seen before in Figure 5A, the hydrogel monomer structure seems to dictate the observed distinct trends of the three systems, as the impact on the extent of swelling (*Q*) is not a unique function of ϕ_eff_. For example, some of the low molecular weight hydrogels (☆) fall on the upper dashed line computed (eq 9) for *Q* = 1 (which would be the relaxed state), whereas the cationic charged gels (Δ) conform to higher *Q* ∼ 4, without the whole group following the same exact trend. For the neutrally charged gels (○), *M*_s_(*G*_s_) follows an intermediate *Q* ∼ 1.5. Overall, the estimation of *G*_s_ from the experimental *M*_s_ data is subject to significant uncertainties due to the inevitable effects of swelling.

## Conclusion

Shear (*G*) and longitudinal (*M*) moduli are biomechanical properties of different physical origin, so care must be taken when assuming that these quantities are universally correlated. Nevertheless, in many practical settings, different underlying biochemical, physical, and structural changes in the samples are found to affect both moduli in the same manner leading to observed empirical correlations. Previously, the equilibrium swelling theory and rubber elasticity theory have been used to describe and understand the mechanical properties and the swelling-to-equilibrium behavior of hydrogels, considering inherent properties of the hydrogels, such as the polymer-monomer interaction parameter, molecular weight between cross-links, molecular weight of the monomer, polymer volume fractions in the relaxed and swollen states, and molar and specific volumes of the solvent.^43,45,46^ It has also been defined that the charge of the monomers in the system, the pH level, and the presence of salt in the system also affect these swelling properties and hence the mechanical properties of the hydrogel after swelling.^38,46−49^ In this work, we employed the rubber elasticity theory and scaling concept of semidilute polymer solutions to describe the global stiffness of a hydrogel, based on some of the inherent characteristics of the monomer and solvent. We also calculated an effective polymer volume fraction to better describe the experimentally measured shear modulus; on the other hand, there is no similar theory to describe the experimentally measured longitudinal modulus.

We proposed understanding the mechanical properties of gels based on an effective polymer volume fraction of each hydrogel. We demonstrated that both the shear *G*_r_ and longitudinal moduli *M*_r_ of a hydrogel depend on the effective polymer volume fraction, ϕ_eff_. Both moduli can be correlated and predicted from each other with necessary assumptions of the hydrogel, namely, the solvency and the Kuhn segment length. Although the correlation is still system-dependent and not universal, we now provide a physical explanation of the correlation.

For hydrogels in the swollen state, the force balance changes and a new explanation of the mechanical properties needs to be considered. Practically, due to the different underlying physics and processes of the different moduli, swelling affects the moduli in the same direction but with different functional forms. Also, in this case, we were able to predict the mechanical behavior of the hydrogel for separate cases. For the shear modulus, the behavior of the polymer chain at the microscopic scale affects the hydrogel at the macroscopic scale of the modulus in a volumetric manner. Alternatively, the longitudinal modulus was affected as an average of the solid and fluid components, and the fit of the effective polymer volume fraction is affected directly by the swelling ratio as *Q*^–1^, following the inverse law of mixtures. It is worth noting that in the swollen case, the shear modulus measurements already display a nonuniversal behavior across different hydrogel formulations, implying that additional information is needed to understand the structure-mechanics relationship in traditional rheology measurements. For the correlation between the two moduli, both are affected by the swelling ratio, determined by the water intake, which also depends on the relaxed polymer volume fraction and the cross-linking density. As a result, a prediction for the moduli of the swollen hydrogels is feasible, provided that information on the relaxed state of the system and the swelling ratio are available. Biological systems are more complex than a controlled hydrogel system, but this work provides fundamental insights as to why empirical correlations have been observed even in such complex systems, i.e., a common dependence of low-frequency shear and high-frequency longitudinal modulus on the different variables intrinsic to the system; however, due to the system-dependent nature of the correlation, an initial calibration should be performed for each specific system.

## Data Availability

All data needed to evaluate the conclusions in the paper are present in the paper and/or the Supporting Information.

## Supporting Information Available

Additional experimental results are presented, including longitudinal modulus *M* vs polymer volume fraction ϕ of solutions; Brillouin line width vs ϕ; comparison of ϕ_Sw_ vs ϕ_Seff_ and analysis of the precision of the assumed ϕ for the swollen hydrogels; prediction of *M* in swollen hydrogels; and analysis of dependence of *Q* and *M* with ϕ_eff_ (PDF)
