# Supplementary material for: Network Viscoelasticity from Brillouin Spectroscopy
Source: Biomacromolecules. 2023 Dec 29;25(2):955–63. doi: 10.1021/acs.biomac.3c01073 (PMC10865340; doi:10.1021/acs.biomac.3c01073)
Supplement: Supplementary file 1 — bm3c01073_si_001.pdf [file bm3c01073_si_001.pdf]

Supplementary information for

Network viscoelasticity from Brillouin spectroscopy.

Authors.
Raymundo Rodríguez-López[1], Zuyuan Wang[2], Haruka Oda[3], Metecan Erdi[4], Peter Kofinas[4], George Fytas[5,6]*, Giuliano Scarcelli[1]*

Affiliations.
[1]Fischell Department of Bioengineering, University of Maryland, College Park, MD 20742, USA.
[2]School of Mechanical and Electrical Engineering, University of Electronic Science and Technology of China, Chengdu, Sichuan 611731, China.
[3]School of Information Science and Technology, The University of Tokyo, Tokyo 113-8656 Japan.
[4]Department of Chemical and Biomolecular Engineering, University of Maryland, College Park, MD 20742, USA.
[5]Max Planck Institute for Polymer Research, Ackermannweg 10, 55128. Mainz, Germany.
[6]Institute of Electronic Structure and Laser, FO.R.T.H, N.Plastira 10, Heraklion, 70013, Grecce

***Corresponding author e-mail:** scarc@umd.edu, fytas@mpip-mainz.mpg.de

This document includes:

Supplementary Figures.
S1. Longitudinal Modulus M vs polymer volume fraction ϕ of polyacrylamide solution mix.
S2. Brillouin Linewidth vs ϕ.
S3. Measured ϕ_{Sw} vs ϕ_{Seff} of Swollen Hydrogels.
S4. A. Longitudinal modulus M vs ϕ_{Seff}; B. Comparison of the measured ϕ_{Sw} vs the assumed $\phi_{SwollenGels}$ when perfect behavior
S5. Experimental vs predicted longitudinal modulus M.
S6. A. Swelling Ratio Q vs ϕ_{eff}; B.Swelling Ratio Q vs $\phi_{Seff}=\phi_{eff}/Q$
S7. The ratio M_S/M_r vs Q in the swollen state

Supplementary Figures

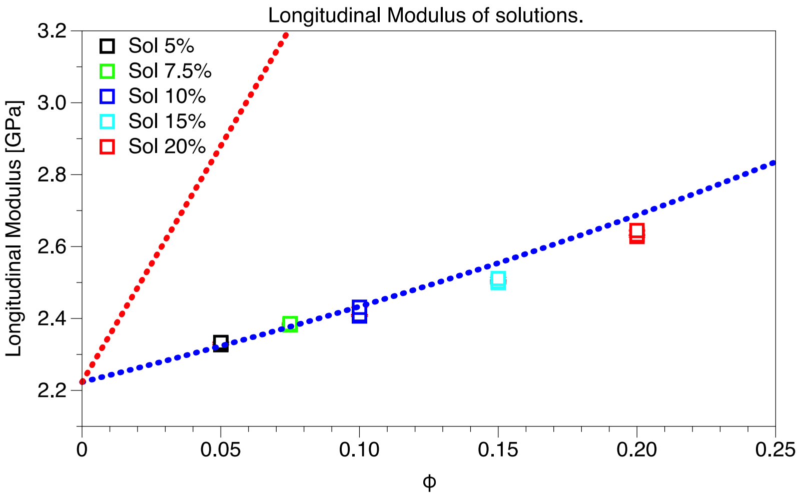

Supplementary Figure S1. Longitudinal Modulus *M* vs polymer volume fraction ϕ of polyacrylamide solution mix. This experimental value gives the sets the expected behavior of the gels because the volume fraction is totally controlled only by the amount of polymer added to the mixture. Linear and inverse law of mixtures follow the red and blue pointed line, respectively.

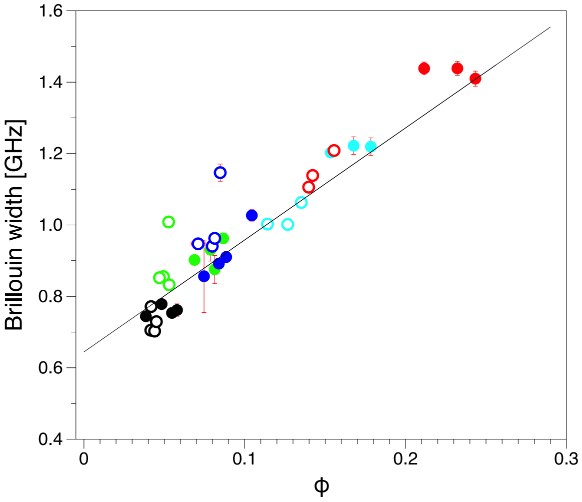

Supplementary Figure S2. Brillouin Linewidth vs ϕ**.** Brillouin linewidth presents a slight increase as ϕ increases, but far from the transition from liquid to solid phase.

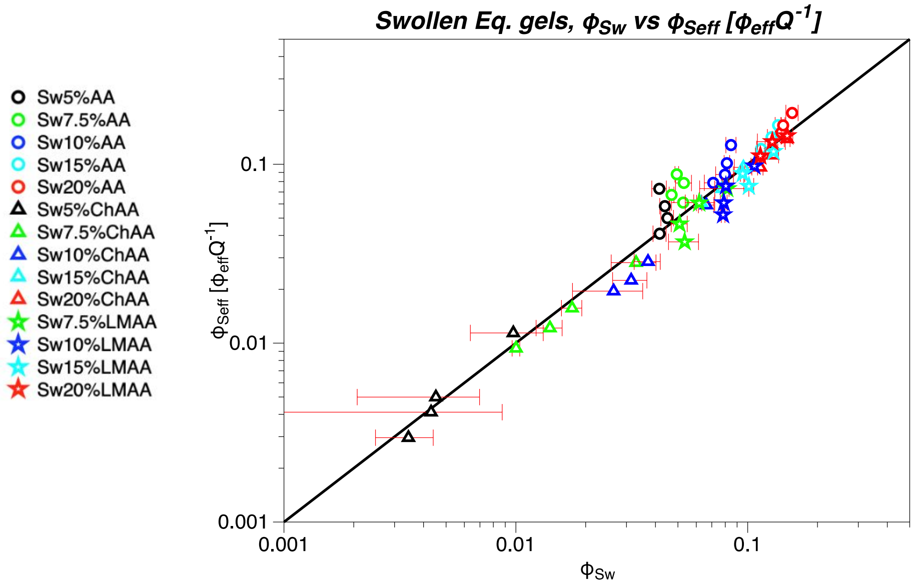

Supplementary Figure S3. Measured ϕ_{Sw} vs ϕ_{Seff} of Swollen Hydrogels. Comparison of the measured ϕ_{Sw} vs the assumed $\phi_{Seff}=\phi_{eff}Q^{-1}$ where ϕ_{eff} refers to the relaxed state and Q is the experimental swelling ratio. Line represents $\phi_{Sw=}\phi_{Seff}$

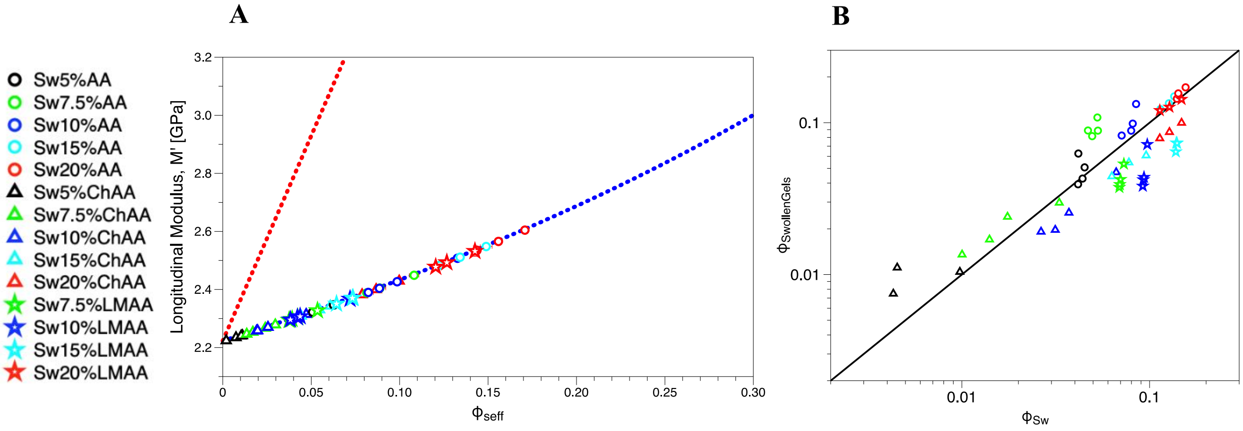

Supplementary Figure S4 A. Longitudinal modulus *M* vs ϕ_{Seff}, as a perfect behavior following the inverse rule of mixtures law. **B**. Comparison of the measured ϕ_{Sw} vs the assumed $\phi_{\mathrm{SwollenGels}}$ when perfect behavior. A lot of scattering suggests that this is not a good fit. Black line represents $\phi_{\mathrm{Sw}} = \phi_{\mathrm{Swollengels.}}$

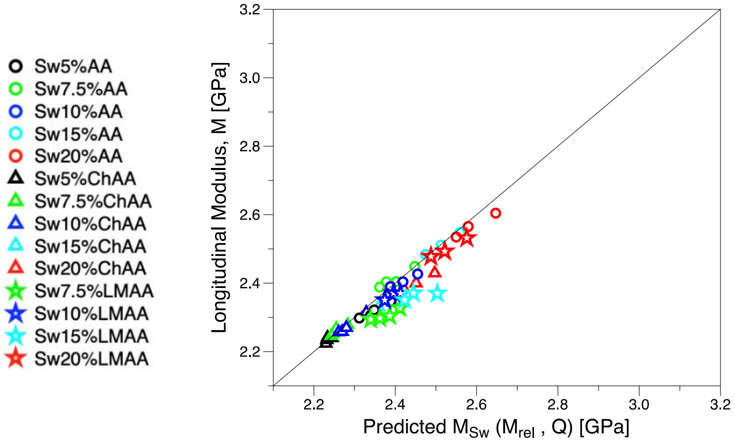

Supplementary Figure S5. Experimental vs predicted longitudinal modulus *M*. Predicted *M* (x axis) is calculated with $\phi_{Seff} = \frac{\phi_{eff}}{Q}$.

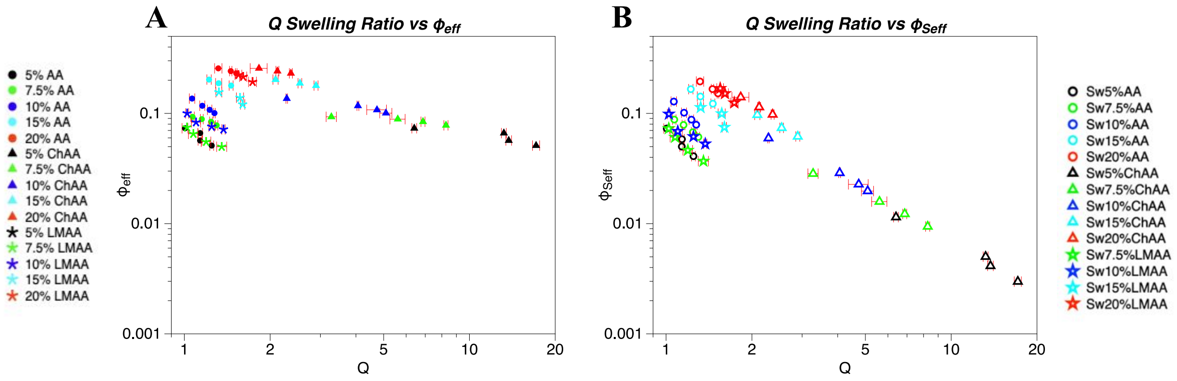

Supplementary Figure S6. **A**. Swelling Ratio vs ϕ_{eff}. Only ChAA shows a superposition of the expected dilution trend upon swelling assuming Q up to ~20. The other two chemistries show not superimposed swelling and Q increases only up ~2 at similar ϕ_{eff}. **B**. Swelling Ratio Q vs $\phi_{Seff}=\phi_{eff}/Q$. This plot expresses the composition of the swollen hydrogels relatively to the relaxed state. The composition in the swollen state expectedly drops stronger with Q, but displaying same pattern with the plot in A. The swelling at similar ϕ_{eff} is system specific.

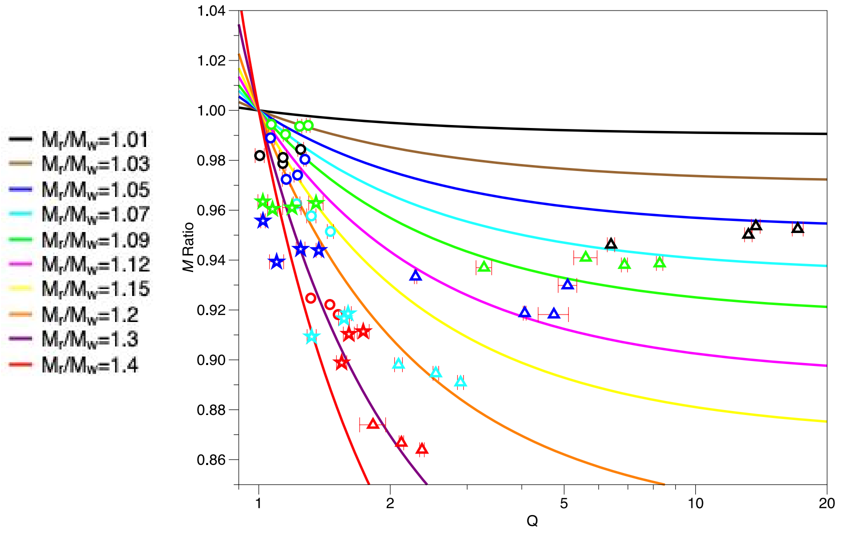

Supplementary Figure S7. The ratio M_S/M_r vs Q (in log scale for higher resolution) in the swollen state. The solid lines represent the inverse rule of mixtures prediction, $M_S/M_r = [(M_r/M_W)-(M_r/M_W-1)/Q]^{-1}$ (Eq.9) at different M_r/M_W values (1.01-1.4 in the color scale). The figure suggests that all swollen hydrogels conform to inverse rule of mixtures law but adjusting M_r/M_W (1.01 to 1.4) or ϕ_{eff} (eq. 4). Compared to the system dependent swelling of Fig.S5, this system specificity can be lifted in the case of M_S only if both Q and ϕ_{eff} are considered. Otherwise M_S (ϕ_{Seff}) is apparently system dependent.
